# Protective Effect of Silymarin on Noise-Induced Hearing Loss in Guinea Pigs

**DOI:** 10.5812/ircmj.8890

**Published:** 2013-11-05

**Authors:** Ghassem Mohammadkhani, Akram Pourbakht, Mahnaz Khanavi, Soghrat Faghihzadeh

**Affiliations:** 1 Department of Audiology, School of Rehabilitation, Tehran University of Medical Sciences, Tehran, IR Iran; 2 Department of Audiology, Rehabilitation Research Center, School of Rehabilitation Sciences, Iran University of Medical Sciences, Tehran, IR Iran; 3 Department of pharmacognosy and Traditional, Iranian Medicine Research Center, School of Pharmacy, Tehran University of Medical Sciences, Tehran, IR Iran; 4 Department of Biostatistics and Social Medicine, Zanjan University of Medical Sciences, Zanjan, IR Iran

**Keywords:** Hearing Loss, Noise-Induced, Silymarin, Auditory Brain Stem Implantation

## Abstract

**Background:**

Hearing capability plays a principal role on human's communication. Noise-induced hearing loss (NIHL) caused by exposure to high noise levels is a serious socio-economic problem in modern societies. NIHL can either be reversible, resulting in a temporary threshold shifts (TTS) or irreversible, resulting in a permanent threshold shifts (PTS). PTS is often confirmed in the time span of between 2 - 6 weeks. NIHL may be prevented by avoidance of excessive amounts of noise or reducing the sound energy entering the inner ear using hearing protective devices. However, there are some conditions that such prevention is not possible such as noise exceeding the protective capabilities of the hearing protection device, working in military or the person does not tolerate the protection device. Thus the protective agent for preventing NIHL would be useful.

**Objective:**

Free radical molecules and consequence oxidative stress have been shown to play a significant role in noise-induced hearing loss. Silymarin is an antioxidant flavonoid complex derived from the herb milk thistle has ability to mitigating the oxidative stress, scavenge free radicals. In the current study, we aimed to evaluate the protective effect of silymarin on noise induced hearing loss in guinea pig by auditory brain stem response.

**Materials and Methods:**

Twenty guinea pigs randomly divided into 2 groups. The animals in the experimental group were intraperitoneally injected with 100 mg/kg/day silymarin dissolved in propylene glycol for 6 consecutive days. The control subjects were intraperitoneally injected with propylene glycol for 6 consecutive days. All animals were exposed to 4 kHz octave band noise at 120 dB SPL for 6 hours. Auditory brainstem responses (ABRs) at frequencies of 2, 4, 6, 8, 12, 16 and 20 kHz were precisely recorded before intervention and then on intervals of 0, 3, 10 and 15 days after noise exposure. Data were analyzed using repeated measures ANOVA.

**Results:**

Threshold shifts for the experimental group at all frequencies immediately, 3, 10 and 15 days after noise exposure were significantly reduced compared to the control group (P < 0.01).

**Conclusions:**

The findings indicate a protective effect of silymarin on temporary and permanent noise-induced hearing loss.

## 1. Background

Hearing capability plays a principal role on human's communication. Noise-induced hearing loss (NIHL) caused by exposure to high noise levels is a serious socio-economic problem in modern societies. NIHL can either be reversible, resulting in a temporary threshold shifts (TTS) or irreversible, resulting in a permanent threshold shifts (PTS). PTS is often confirmed in the time span of between 2 - 6 weeks (1, 2). NIHL may be prevented by avoidance of excessive amounts of noise or reducing the sound energy entering the inner ear using hearing protective devices. However, there are some conditions that such prevention is not possible such as noise exceeding the protective capabilities of the hearing protection device, working in military or the person does not tolerate the protection device. Thus the protective agent for preventing NIHL would be useful. Several studies have demonstrated that NIHL causes the production of reactive oxygen species (ROS) such as hydroxyl radicals, reactive nitrogen species (RNS) such as peroxynitrite, and other free radical molecules in the cochlea (3-5). NIHL also causes the increase of endogenous antioxidants such as glutathione that protect the cells against oxidative stress (6). The oxidative compounds directly destroy DNA and cell membranes leading hair cell damage (7, 8). Therefore, antioxidants that detoxify these free radicals may serve to protect or rescue hair cells damage (9-14). One such agent is silymarin, an antioxidant flavonoid complex derived from the herb milk thistle (silybum marianum) (15). It has been used for treatment of liver diseases and prevention of cancer (16). These properties seem to be due to its ability to mitigating the oxidative stress, scavenge free radicals and to chelate metal ions (17). Nabila et al. (2010) reported that silymarin protects against acute cisplatin nephrotoxicity and may be considered potentially useful candidate in the combination with chemotherapy by acting in the kidney as a potent scavenger of free radicals thus preventing the toxic effect of cisplatin both the histological and ultrastructural levels (18).

Since oxidative stress is involved in the mechanism of NIHL, and in accordance with latter reports that silymarin has antioxidant properties, the aim of this study was to evaluate the protective capacity of silymarin on noise induced hearing loss in guinea pig by auditory brain stem response.

## 2. Objective

Free radical molecules and consequence oxidative stress have been shown to play a significant role in noise-induced hearing loss. Silymarin is an antioxidant flavonoid complex derived from the herb milk thistle has ability to mitigating the oxidative stress, scavenge free radicals. In the current study, we aimed to evaluate the protective effect of silymarin on noise induced hearing loss in guinea pig by auditory brain stem response.

## 3. Materials and Methods

Twenty male six weeks old albino guinea pigs (290 ± 10g) were prepared from Pasteur Institute (Tehran, Iran). Since sex differences have been associated with a deferring ability to detoxify ROS, only male guinea pigs were used (19). The subjects were housed with free access to water and food in their cages. Temperature was maintained at 20 ± 2oc. Lights were on from 7.00 am to 7.00 pm (12:12 h light: dark cycle). All procedures regarding care and use of guinea pigs reviewed and approved by ethics committee of Tehran University of Medical Sciences. Before experiment, animals kept in a quiet room for 3 days for adapting to the new living condition. ABRs were performed for all subjects before intervention using Biologic Navigator pro system (Natus, USA). With this system a custom stimulus can be used for the auditory stimulus output. The custom stimulus lets we use a WAV file as the stimulus presented via the selected transducer with the intensity level and rate controlled by the program. To use the custom stimulus feature the protocol stimulus type must be set to "Custom" and the desired WAV file is selected. Bio-logic does not provide wav files. We have to provide our own wav files by MATLAB software. The custom sound stimulus consisted of a 5 ms tone burst at frequencies of 2, 4, 6, 8, 12, 16 and 20 kHz in the WAV format. The output of transducer was measured in SPL and additional calibration correction value for each individual WAV file was stored in an ASCII (text) file with the same file name as the WAV file but with the extension of nrm. Animals were anesthetized with a mixture of xylazine (10 mg/kg) and ketamine (40 mg/kg) given intramuscularly, and normal body temperature was maintained with a heating pad. An inverting needle electrode was placed subcutaneously below the test ear and a non-inverting electrode at the vertex. A ground electrode was positioned below the contralateral ear. The electrical responses from the recording electrodes were amplified (× 100000), filtered (100-3000 Hz). The sound intensity was varied in 10 dB steps down and then 5 dB steps up near threshold. One thousand and twenty-four tone presentations delivered at the rate of 23.1 were averaged to obtain a waveform at each level. Hearing threshold was defined as the lowest level at which a clear ABR peaks 3 can be detected. Replications were obtained at stimulus levels around threshold.

**Figure 1.**
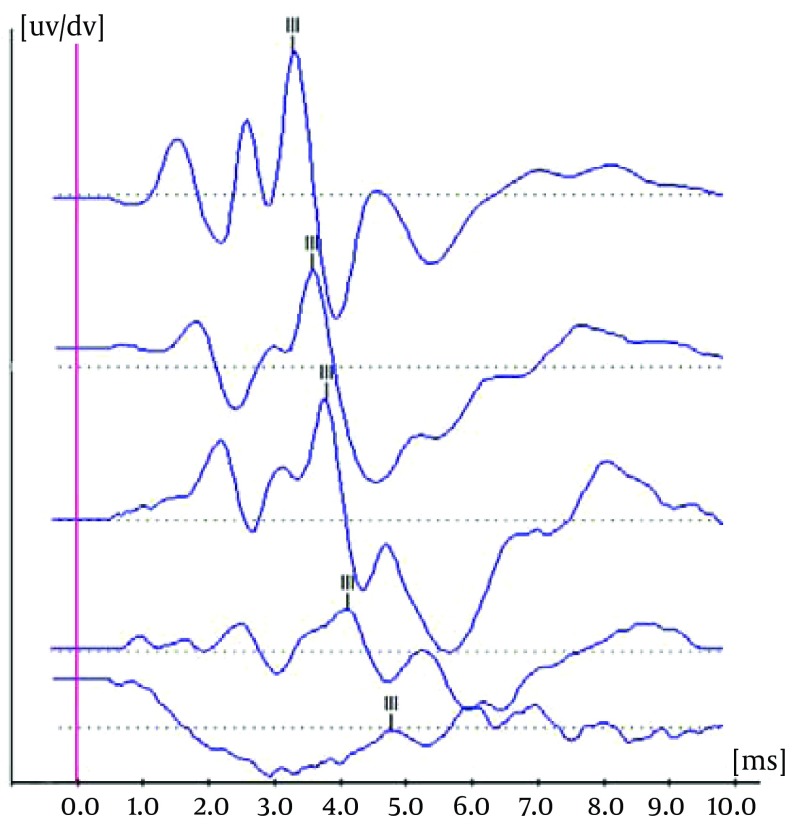
Sample of ABR Thresholds in Guinea Pig

After baseline ABR threshold measurements animals were randomly divided into two groups (n = 10, each). In the experimental group, subjects were i.p. injected for 6 consecutive days with silymarin dissolved in propylene glycol at a dose of 100 mg/kg/day. In the control group, the animals were i.p. injected for six consecutive days with propylene glycol. One hour after the final intervention each animal was separately exposed for six hours to 4 kHz octave band noise at 120 dB SPL. Noise exposure was carried out in a lighted and ventilated anechoic chamber while animals having free access to food and water. The chamber was ﬁtted with speakers driven by a noise generator and a power ampliﬁer. Sound levels were measured and calibrated at the location of animal cage by a B&K 2231 sound level meter. Immediately, 3, 10 and 15 days after noise exposure, ABR thresholds were repeated. Sample of ABR thresholds in guinea pig were shown in Figure 1. 

ABR thresholds were analyzed by repeated measures ANOVA in both groups at all test frequency in 5 days (pre-exposure, immediately, 3, 10 and 15 days after noise exposure). Statistical comparisons were made using the SPSS 17 software package and P < 0.01 was statistically considered significant.

## 4. Results

Baseline ABR thresholds at 2, 4, 6, 8, 12, 16 and 20 kHz were observed as displayed on Table 1. There were no significant differences in baseline ABR thresholds between groups. 

**Table 1.** Baseline auditory brainstem responses (ABRs) Thresholds in Study Groups (n = 10, each)

Frequency, Hz	2000	4000	6000	8000	12000	16000	20000
**Control, Mean (SD)**	27.50 (2.63)	19.5 (2.83)	14.50 (3.68)	9.50 (2.83)	6.00 (3.16)	4.50 (4.11)	2.50 (4.24)
**Experimental, Mean (SD)**	26.50 (4.11)	20.00 (3.33)	15.00 (4.08)	9.00 (3.16)	5.50 (4.37)	4.00 (2.10)	3.50 (2.41)

**Figure 2.**
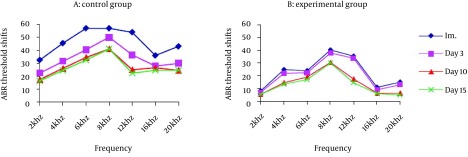
Mean ABR Threshold Shifts Immediately, 3, 10 and 15 Days after Noise Exposure; A: Control Group. B: Experimental Group.

Hearing loss was observed immediately after noise exposure in all subjects. As shown in Figure 2, immediately after exposure, mean noise produced temporary threshold shifts ranging from 30 dB at 2 kHz to 57 dB at 6 kHz in control group. In experimental group, the minimum and maximum threshold shifts were 9.5 dB at 2 kHz and 40.5 dB at 8 kHz, respectively. Data showed that silymarin reduced the TTS up to 32 dB compared to control group. The mean of ABR thresholds in experimental subjects were better than control group at all test frequencies. Statistical analysis using repeated measures ANOVA showed significant differences in temporary threshold shifts at all frequencies between two groups (P = 0.00). 

In two groups recovery of threshold shifts were occurred over time; however, the degree of recovery differed among the groups. Fifteen days after noise exposure, ABR thresholds shifts were ranging from 16.5 dB at 2 kHz to 41 dB at 8 kHz in control group. In experimental group, the minimum and maximum threshold shifts were 5 dB at 20 kHz and 30 dB at 8 kHz, respectively. Data indicated that silymarin had reduced the PTS up to 19 dB compared to control group. Hearing loss in experimental group was less than control group 15 days after noise exposure. These differences were statistically significant (P = 0.00).

## 5. Discussion

In this study, guinea pigs were exposed to 4 kHz octave band noise at 120 dB SPL for six hours to evaluate the protective capacity of silymarin on TTS and PTS. The results indicated that this antioxidant agent could effectively attenuate the temporary and permanent noise-induced ABR threshold shift in guinea pigs. The ABR thresholds showed that silymarin reduced the TTS up to 32 dB and PTS up to 19 dB compared to control group. When we observed the ABR threshold shifts at different time scales, we concluded that the recovery was greater when silymarin pretreatment implemented. Therefore, data from current study indicated that silymarin is capable to protect the cochlea from noise-induced hearing loss. The cochlear hair cell damage due to ROS and/or RNS production following loud sound exposure has already been demonstrated (3-5). It is assumed that NIHL can be prevented with antioxidants that detoxify these free radicals (9-14). Silymarin, an antioxidant flavonoid complex derived from the herb milk thistle (15), is frequently used in the treatment of liver diseases. It is capable of protecting liver cells directly by stabilizing the membrane permeability through inhibiting lipid peroxidation (20) and preventing liver glutathione depletion (21). Silymarin possesses inhibiting lipid peroxidation, membrane stabilizing, anti-inflammatory, antioxidant, RNA, protein and DNA synthesis stimulating properties (20, 22-24). In addition, silymarin studies on human demonstrated no significant adverse reaction (25). Since NIHL has also been attributed to DNA oxidation and lipid peroxidation in the cochlea (26, 27), silymarin could specifically protect the temporary and permanent cochlear hair cell loss. In the current study, we had considerable TTS immediately after noise exposure to 4 kHz octave band of noise at all frequencies measured. The prominent TTS were seen at 6, 8, and 12 kHz. It can be explained by the highest energy level of 4 kHz octave band of noise which occur half or one octave above the center frequency. But silymarin attenuated the extent of ABR threshold shifts more significantly at 6, 16 and 20 kHz than other frequencies immediately after noise exposure. In other words silymarin was more effective at the frequencies differ from the frequencies with greater threshold shift. Such condition that an antioxidant was more effective at the frequencies away from the frequencies with greater threshold shift has been reported in other studies. Ohinata et al. (2000) reported that 4 kHz octave band noise produced greater threshold shifts at 4, 8, and 12 kHz than at 2, 4, and 16 kHz in guinea pigs, but glutathione supplementation attenuated the extent of threshold shifts more prominently at 2, 4, and 16 kHz. Such phenomenon could possibly be verified that other factors, in addition to ROS formation, may have contributed to maximum threshold shifts (26). Additionally, it can be occurred because the uptake of medicine is higher at the base of cochlea. Despite the outstanding progress in hearing conservation programs (HCPs) made-approaches for reducing noise and improving hearing protector devices-noise induced cochlear injury remains a common and costly problem. Pharmacological intervention with antioxidant agents such as silymarin may be used as a complementary to HCPs, but may not replace it. The pharmacological approaches could fix some of the limitations of HCPs likewise conditions that the noise hazard exceeds the protective capability of hearing. In such condition an otoprotective agent could be taken to supplement protection. The result of this study indicated that silymarin is capable to protect the cochlea from temporary and permanent noise induced hearing loss. Data from the current study open promising possibility for the clinical application of silymarin to protect the ear from NIHL. It is important to note that the efficacy of taking this agent need to be confirmed in scientifically and ethically performed clinical trials.
